# Analysis of age-related changes in psychosine metabolism in the human brain

**DOI:** 10.1371/journal.pone.0193438

**Published:** 2018-02-26

**Authors:** Michael S. Marshall, Benas Jakubauskas, Wil Bogue, Monika Stoskute, Zane Hauck, Emily Rue, Matthew Nichols, Lisa L. DiAntonio, Richard B. van Breemen, Jeffrey H. Kordower, Carlos A. Saavedra-Matiz, Ernesto R. Bongarzone

**Affiliations:** 1 Department of Anatomy and Cell Biology, College of Medicine, University of Illinois at Chicago, Chicago, IL, United States of America; 2 Department of Medicinal Chemistry and Pharmacognosy, College of Pharmacy, University of Illinois at Chicago, Chicago, IL, United States of America; 3 Division of Genetics, Newborn Screening Program, Wadsworth Center, New York State Department of Health, Albany, NY, United States of America; 4 Department of Neurological Sciences, Rush University Medical Center, Chicago, IL, United States of America; 5 Departamento de Química Biologica, Facultad de Farmacia y Bioquímica, Universidad de Buenos Aires, Buenos Aires, Argentina; Northeastern Ohio Medical University, UNITED STATES

## Abstract

α-Synuclein aggregation has been linked to Gaucher’s disease (GD) and Krabbe’s disease (KD), lysosomal conditions affecting glycosphingolipid metabolism. α-Synuclein pathology has been directly attributed to the dysregulation of glycosphingolipids in both conditions, specifically to increased galactosylsphingosine (psychosine) content in the context of KD. Furthermore, the gene (*GALC*) coding for the psychosine degrading enzyme galactosylceramidase (GALC), has recently been identified as a risk loci for Parkinson’s disease. However, it is unknown if changes in psychosine metabolism and GALC activity in the context of the aging human brain correlate with Parkinson’s disease. We investigated psychosine accumulation and GALC activity in the aging brain using fresh frozen post-mortem tissue from Parkinson’s (PD, n = 10), Alzheimer’s (AD, n = 10), and healthy control patients (n = 9), along with tissue from neuropsychiatric patients (schizophrenia, bipolar disorder and depression, n = 15 each). An expanded mutational analysis of PD (n = 20), AD (n = 10), and healthy controls (n = 30) examined if PD was correlated with carriers for severe *GALC* mutations. Psychosine content within the cerebral cortex of PD patients was elevated above control patients. Within all patients, psychosine displayed a significant (p<0.05) and robust regional distribution in the brain with higher levels in the white matter and substantia nigra. A mutational analysis revealed an increase in the incidence of severe *GALC* mutations within the PD patient population compared to the cohorts of Alzheimer’s patients and healthy controls tested. In addition to α-synuclein pathology identified in the KD brain, control patients identified as GALC mutational carriers or possessing a GALC pathogenic variant had evidence of α-synuclein pathology, indicating a possible correlation between α-synuclein pathology and dysregulation of psychosine metabolism in the adult brain. Carrier status for *GALC* mutations and prolonged exposure to increased psychosine could contribute to α-synuclein pathology, supporting psychosine metabolism by galactosylceramidase as a risk factor for Parkinson’s disease.

## Introduction

A growing body of evidence underlines the role lysosomal dysfunction contributes to the pathology in several neurological conditions, including demyelination, late-onset neurodegenerative diseases, and aging [1, 2]. For example, a robust link has been described between Parkinson’s disease (PD) and Gaucher’s disease [3–5], a lysosomal sphingolipidosis caused by mutations in the glucocerebrosidase (*GBA1*) gene. *GBA1* mutations have been identified as one of the strongest risk factors for PD and are associated with an earlier onset of symptoms, increased levels of α-synuclein pathology in neurons, and greater cognitive decline [3, 6, 7].

We described proteopathic aggregations of fibrillized α-synuclein in central neurons of infantile and young adult patients affected by Krabbe’s disease (KD), a lysosomal sphingolipidosis caused by mutations in the galactosylceramidase (*GALC*) gene [8], describing for the first time a link between GALC metabolism and synucleinopathies [9]. Deficiency of lysosomal GALC activity in patients homozygous for Krabbe mutations leads to the rapid accumulation of galactosylsphingosine (or psychosine), a neurotoxic sphingolipid to neurons and myelinating cells [10–17]. *In vitro*, we found that psychosine is also a compound that potently accelerates aggregation of α-synuclein [8], by direct binding to the carboxy-terminus and facilitation of an aggregation-prone protein conformation (Abdelkarim, Marshall, Gaponenko & Bongarzone, under review). Additionally, *GALC* has been identified as a risk loci for Parkinson’s disease, further supporting a connection between PD and GALC mediated psychosine metabolism [18]. Whether alterations in the psychosine metabolism resulting from inefficient lysosomal function could contribute to α-synuclein pathology in the adult human brain during aging is currently unknown.

To investigate this possibility, here we examined whether psychosine degradation is affected by aging in the human brain, and whether these changes were associated with mutations and/or small polymorphisms (SNPs) in the *GALC* gene. For this, we studied post-mortem brain tissue for psychosine content and GALC activity during aging in healthy control individuals compared to patients diagnosed with PD and Alzheimer’s disease (AD). Changes were also measured in a neurological control cohort comprised of patients diagnosed with schizophrenia, bipolar disorder and depression. Psychosine was found to have a robust distribution within brain regions, particularly concentrated in the white matter of the cortex and the substantia nigra. Psychosine content within the cortex of PD patients was found to be elevated in comparison to healthy control tissue, and psychosine increases and GALC activity reduction were shown to be correlated with aging. The mutational analysis for GALC SNPs found 10% (2/20) of PD patients tested to be a carrier for a severe *GALC* mutation compared to 0% (0/10) of Alzheimer’s patients and 6.67% (2/30) of healthy controls. However, under histological examination, both control individuals were positive for Lewy body-like inclusions, potentially identifying a non-symptomatic synucleinopathy such as incidental Lewy body disease.

These results show for the first time that during normal aging, psychosine metabolism is progressively dysfunctional, leading to the accumulation of this lipid in the aging brain, especially in regions vulnerable to degeneration in PD. These results underline the possibility that defects in the metabolism mediated by changes in GALC activity modify the risk of developing α-synuclein pathology in vulnerable patients.

## Materials and methods

### Tissue

Fresh-frozen issue was obtained from the following brain banks: Harvard Brain Tissue Resource Center, University of Maryland Brain and Tissue Bank, Human Brain and Spinal Fluid Resource Center at UCLA, for the neurodegenerative cohort, and Stanley Medical Research Institute provided samples that were used in the neuropsychiatric cohort analysis. Fixed tissue from additional PD patients was obtained from Dr. Kordower at Rush University to be used in the DNA mutational analysis screening. This work was performed under UIC guidelines and samples de-identified and therefore exempt from an IRB full protocol. Fresh-frozen tissue to be used for biochemical analysis was microdissected using a sterile scalpel, separating white matter from gray within the frontal cortex, and tissue from the substantia nigra and caudate. Tissue was kept frozen on dry ice during dissections.

### Mass spectrometry

Psychosine measurements were performed as previously described [8]. Briefly, fresh frozen tissue was homogenized in H_2_O using a Vibra-cell ultrasonic liquid processor model#VCX 130 (Sonics and Materials Inc., Newton, CT). Psychosine was extracted from tissue homogenates (200 μg) via a methanol-acetic acid solution (0.5% Acetic Acid in methanol). Using D-lactosyl-β1–1'-D-*erythro*-sphingosine cat#860542P (Avanti Polar Lipids, Alabaster, AL) as an internal standard, psychosine content was determined using tandem mass spectrometry. Separations were carried out using a Shimadzu (Kyoto, Japan) Nexera UHPLC system equipped with a Waters Acquity UPLC BEH amide column (2.1 mm X 50 mm, 1.7 μm). The UHPLC system was interfaced to a Shimadzu LCMS-8050 triple quadrupole mass spectrometer equipped with positive ion electrospray and operated at unit resolution. The presence of glucopsychosine was evaluated in lipid extracts from substantia nigra from PD (n = 10), AD (n = 10), non PD controls (n = 10) and KD (n = 3) using positive ion electrospray tandem mass spectrometry with selected reaction monitoring as well as product ion scan as described [14]. Analysis was performed using a Shimadzu LCMS-8050 triple quadrupole mass spectrometer equipped with a Shimadzu Nexera UHPLC system. Psychosines were separated using on a Supelcosil ABZ+ Plus HPLC column (25cm x 4.6mm, 5μm) isocratic 77% methanol, ammonium formate buffer.

### Galactosylceramidase activity

GALC activity measurements were performed from fresh frozen tissue that was homogenized in H_2_O using a Vibra-cell ultrasonic liquid processor model#VCX 130 (Sonics and Materials Inc., Newton, CT). Tissue lysates (20 μg) were incubated with fluorescent GALC substrate (6HMU-beta-D-galactoside; Moscerdam Substrates) for 17 hr at 37°C before the reaction was stopped. Enzymatic activity was assessed via fluorescence using a Beckmann Coulter DTX 880 multimode detector (Beckman Coulter, Brea, CA) using excitation/emission wavelengths of 385 nm and 450 nm, respectively.

### Western-blotting analysis

Protein extracts from SN and cortex were loaded (25μg of protein per sample) into 1.5 mm-10 well 4–12% Bis-Tris Protein Gels. Following gel electrophoresis, proteins were transferred onto PVDF membranes. Membranes were blocked at room temperature for 1 hour in 5% milk in TBS, followed by a TBS wash (5 minutes). Primary antibodies anti-tyrosine hydroxylase, TH (cat. # P40101-0, Pel-Freeze Biological Division, AR) and anti-actin, (cat. # 4967, Cell Signaling, MA) were incubated overnight at 4°C diluted 1:1000 in 1% BSA in TBS. Membranes were washed in TBS three times for 10 minutes. Secondary antibodies were incubated for 1 hour at room temperature and diluted 1:15,000 in 1% milk in TBS-Tween 20 (TBS-T). Membranes were washed in TBS-T three times for 10 minutes, then in TBS for 5 minutes. Blots were visualized on an Odyssey CLx Imaging system. Images were quantitated and plotted using Prism software (Graphpad Software Inc., La Jolla, Ca).

### DNA mutational analysis

DNA was extracted from tissue using the DNeasy Blood and Tissue Kit cat# 69506 (Qiagen, Germantown, MD). Extraction was performed per manufacturer’s protocol. The promoter region, all 17 exons and the intron/exon boundaries of the GALC gene (NG_011853.2) on chromosome 14q31.3 were Sanger sequenced as described [19, 20]. Gap-PCR deletion assays were used to detect the g.30 (most common) and g.7.4 kb deletions. Variants are listed with reference to NM_000153.2 and NP_000144.2 using current HGVS numbering (upstream initiator as codon/1685 amino acid protein).

### Immunohistochemistry

Immunohistological staining was performed using a VECTASTAIN Elite ABC HRP Kit cat# PK-6102 (Vector Laboratories, Burlingame, CA). Staining protocol was performed per manufacturer’s instructions. Primary antibody used was an α-synuclein monoclonal mouse antibody cat# MA1-90342 at 1:500 dilution (ThermoFisher Scientific, Waltham, MA). 3,3'-Diaminobenzidine (DAB) (Sigma-Aldrich, St. Louis, MO) was used as horseradish peroxidase substrate.

### Statistical analysis

Statistics and graphs were prepared with Prism 8 software (Graphpad Software Inc., La Jolla, Ca). Raw data are collated in S1 Table. Data were analyzed using a one-way ANOVA with Gaussian distribution, and the Tukey’s multiple comparison test performed with p-values <0.05 considered significant. Linear regression was considered significant if slope was significantly non-zero. Graphs represent the mean of independent measurements with errors bars representing standard error of the mean. Psychosine measurements were run twice via mass spectrometry analysis to confirm results.

## Results

### Psychosine accumulation and GALC activity follow a robust brain region distribution, with psychosine enriched in the parkinsonian cortex

Post-mortem brain tissue was obtained from various brain depositories and analyzed as two separate age-matched cohorts (Table 1). A neurodegenerative cohort was comprised of age-matched patients diagnosed with PD, AD, or as healthy controls, along with tissue from infantile and late-onset KD patients as positive controls of psychosine lipidosis. Regions available for analysis within this group included white and gray matter of the frontal cortex, substantia nigra and caudate tissue.

**Table 1:** Patient demographics.

Neurodegenerative Cohort			
**Diagnosis**	**N**	**Age (Mean ± SD)**	**Sex**
Infantile Krabbe	2	1.1 ± 0.4	2 Male
Late onset Krabbe	1	39.3	1 Male
Parkinson's	10	77.8 ± 6.5	4 Female, 6 Male
Alzheimer's	10	81.6 ± 8.2	6 Female, 4 Male
Control	9	79.4 ± 9.3	1 Female, 8 Male
Neuropsychiatric Cohort			
**Diagnosis**	**N**	**Age (Mean ± SD)**	**Sex**
Schizophrenia	15	44.5 ± 13.1	6 Female, 9 Male
Bipolar	15	42.3 ± 11.7	6 Female, 9 Male
Depression	14	45.9 ± 9.3	6 Female, 8 Male
Control	15	48 ± 10.7	6 Female, 9 Male
Additional patients for mutational analysis			
**Diagnosis**	**N**	**Age (Mean ± SD)**	**Sex**
Parkinson's	10	67.6 ± 18.6	5 Female, 5 Male
Control	6	66.2 ± 17.3	2 Female, 4 Male

Two cohorts were used for biochemical analysis in this study. The first (neurodegenerative cohort) consisted of aged individuals diagnosed with Parkinson’s or Alzheimer’s disease, along with age-matched healthy controls. Additional tissue from Krabbe patients was included for comparison of pathological changes in psychosine and GALC activity observed in Krabbe’s disease. The second cohort (neuropsychiatric) consisted of middle-age individuals diagnosed with schizophrenia, bipolar disease, or depression, along with age-matched healthy controls.

**Fig 1:**
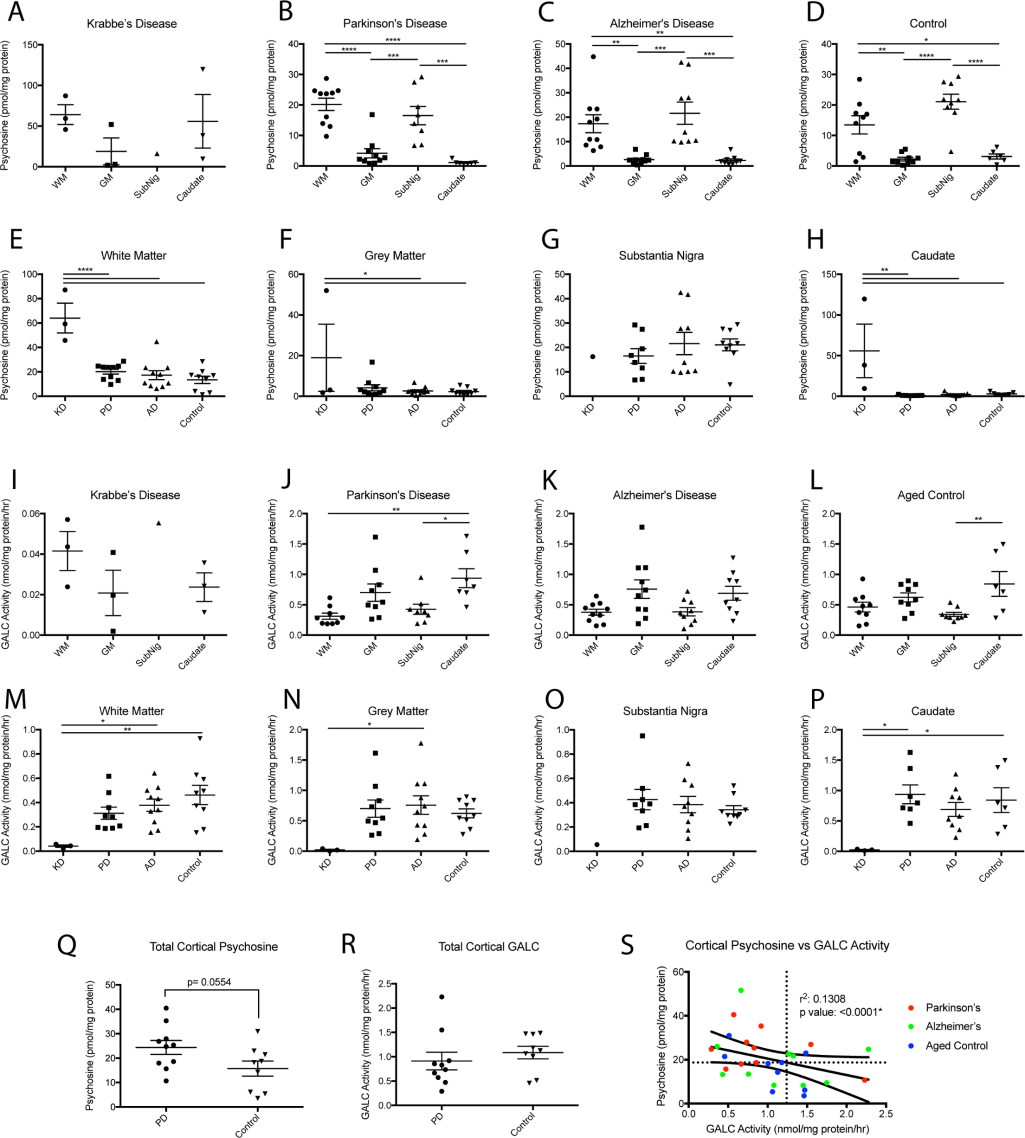
Psychosine content and GALC activity in neurodegenerative cohort. Psychosine concentration in white (WM) and gray (GM) cortical matter, substantia nigra, and caudate was examined in patients diagnosed with Parkinson’s, Alzheimer’s, Krabbe, or healthy controls. A-D) Psychosine was found to have a robust distribution, elevated in the white matter and substantia nigra of all patients. E-H) Psychosine in white and gray matter of Parkinson’s brains trended upwards compared to AD and healthy controls but did not reach significance. GALC activity in white (WM) and gray (GM) cortical matter, substantia nigra, and caudate was examined in patients diagnosed with Parkinsons’s, Alzheimer’s, Krabbe’s, or healthy controls. I-L) GALC activity was found to have a robust distribution, with lower levels found in the white matter and substantia nigra of all patients. M-P) GALC activity in white matter of Parkinson’s brains trended downwards compared to AD and healthy controls but did not reach significance. Q) Total cortical psychosine (summation of white and gray matter of cortex) found higher levels (p = 0.0554) of psychosine in Parkinson’s tissue compared to healthy controls. R) A total cortical GALC activity (summation of white and gray matter of cortex) did not find significantly different levels of GALC activity in Parkinson’s tissue compared to healthy controls. S) Correlation of psychosine to GALC activity as measured within the total cortical tissue of each group. (panel A-P: one way ANOVA with Tukey’s multiple comparison test, *p<0.05. **p<0.01, ***p<0.001, ****p<0.0001, panel Q,R: t-test).

Within the adult brain, psychosine was found to have a robust distribution with significantly higher levels found in the white matter (WM) of the cerebral cortex and the substantia nigra (Fig 1B–1D) compared to the gray matter (GM) of the cerebral cortex and caudate region. This same distribution was not as clear within the Krabbe brain (Fig 1A) potentially due to the small sample size and differences between infantile and adult myelination. When comparing brain regions in the different disease states, the level of psychosine within the white and gray matter had a higher mean within the PD brain as compared to AD and healthy controls but did not reach statistical significance (Fig 1E and 1F). This trend was not observed in the substantia nigra, although similar trends could be obscured by the loss of dopaminergic neurons in the substantia nigra of PD brains, absent in the other disease states and controls. The levels of dopaminergic neurons were estimated by immunoblotting for the dopamine rate-limiting enzyme TH in SN samples from the neurodegenerative cohort. While the levels of dopaminergic neurons in PD patients were lower (S1A Fig), there was no significant correlation between psychosine content and TH levels (S1B Fig). However, measurements of psychosine levels in the brain are limited to post-mortem tissue which precludes an understanding of pre-clinical psychosine levels that may be different at a post-mortem time-point due to neurodegeneration of affected neurons. Last, the caudate was found to be a region that contained negligible amounts of psychosine normally, except in affected Krabbe patients (Fig 1G and 1H). Mass spectrometry analyses for the differential identification of glucopsychosine (glucosyl-sphingosine) and psychosine (galactosyl-sphingosine) detected psychosine as the only species present in all samples from PD, AD and KD cases (S2 Fig).

Tissue samples were also analyzed for GALC activity, which found an inverse distribution compared to the distribution of psychosine (Fig 1I–1P). The difference in GALC activity between brain regions was not as robust as that measured for psychosine. In PD, the GALC activity measured in the caudate was significantly higher than cortical white matter and the substantia nigra levels, though elevations in gray matter did not reach significance. Additionally, the AD brain displayed the same pattern of GALC activity distribution but was not statistically significant between any brain regions. In healthy controls, GALC activity was significantly higher in the caudate compared to the substantia nigra but not in comparison to the cortical white matter (Fig 1I–1L). Distribution within the few samples of Krabbe brain limited these measures as significant. Comparing GALC activity between disease states did not find any significant changes besides the expected complete loss of GALC activity within the Krabbe brain (Fig 1M–1P). GALC activity within the white matter of PD brains did trend lower than AD and healthy controls but did not reach significance, (Fig 1M–1P)

A summation of white and gray cortical psychosine into a total cortical psychosine measurement, found a psychosine elevation in the PD brain that did not meet statistical significance compared to healthy control brains (p = 0.0554, Fig 1Q). We find this comparison relevant to report due to it’s potential biological relevance, especially in light of *GALC*’s identification as a risk foci for PD and because of the growing line of evidence of heterogeneity among patients with PD [21–24]. Statistical significane was likely limited by the sample size available for this study. Total cortical GALC activity was lower than healthy controls, but not significantly and not to the same magnitude of psychosine elevation (Fig 1R). This could indicate that psychosine accumulation with PD occurs via a mechanism other than reduced GALC activity such as age-related generalized lysosomal dysfunction, but could also represent a more limited range that GALC activity deviates within cells.

To clarify this relationship between psychosine and GALC activity, the total cortical psychosine was plotted against GALC activity for each group (Fig 1S). The median psychosine level (18.671 pmol/mg protein) and the corresponding level of GALC activity (1.24 nmol/mg protein/hr) as determined via the best fit curve are indicated with horizontal and vertical lines respectively. These serve to divide the date into 4 quadrants. Overall, the data show a strong negative association between psychosine content and GALC’s activity when all groups are plotted together (a relationship that holds for each group when also plotted individually, data not shown). However, while a large heterogeneity in psychosine’s and GALC’s distribution can be appreciated in each group, the majority of samples from PD patients fell within the upper left quadrant representing individuals with both low GALC and high psychosine, while the majority of aged control samples fell within the bottom two quadrants made up of individuals with psychosine content below the median measurement for the population as a whole. Psychosine accumulation’s inverse relationship to GALC activity has been appreciated in pediatric populations for decades, but this represents the first analysis to our knowledge of its dynamics in the aging human brain.

### Psychosine and GALC activity are distributed similarly in brains of neuropsychiatric disease patients as late-onset neurodegenerative patients but show no pathologic psychosine accumulation

A younger cohort of neurological patients diagnosed with neuropsychiatric illnesses (schizophrenia, bipolar disease, and depression) without α-synuclein pathology and their healthy age-matched controls was analyzed separately to verify any alterations to psychosine and GALC activity outside the context of traditional neurodegenerative diseases. Only white and gray cortical matter from the pre-motor cortex was available for analysis within this second cohort.

**Fig 2:**
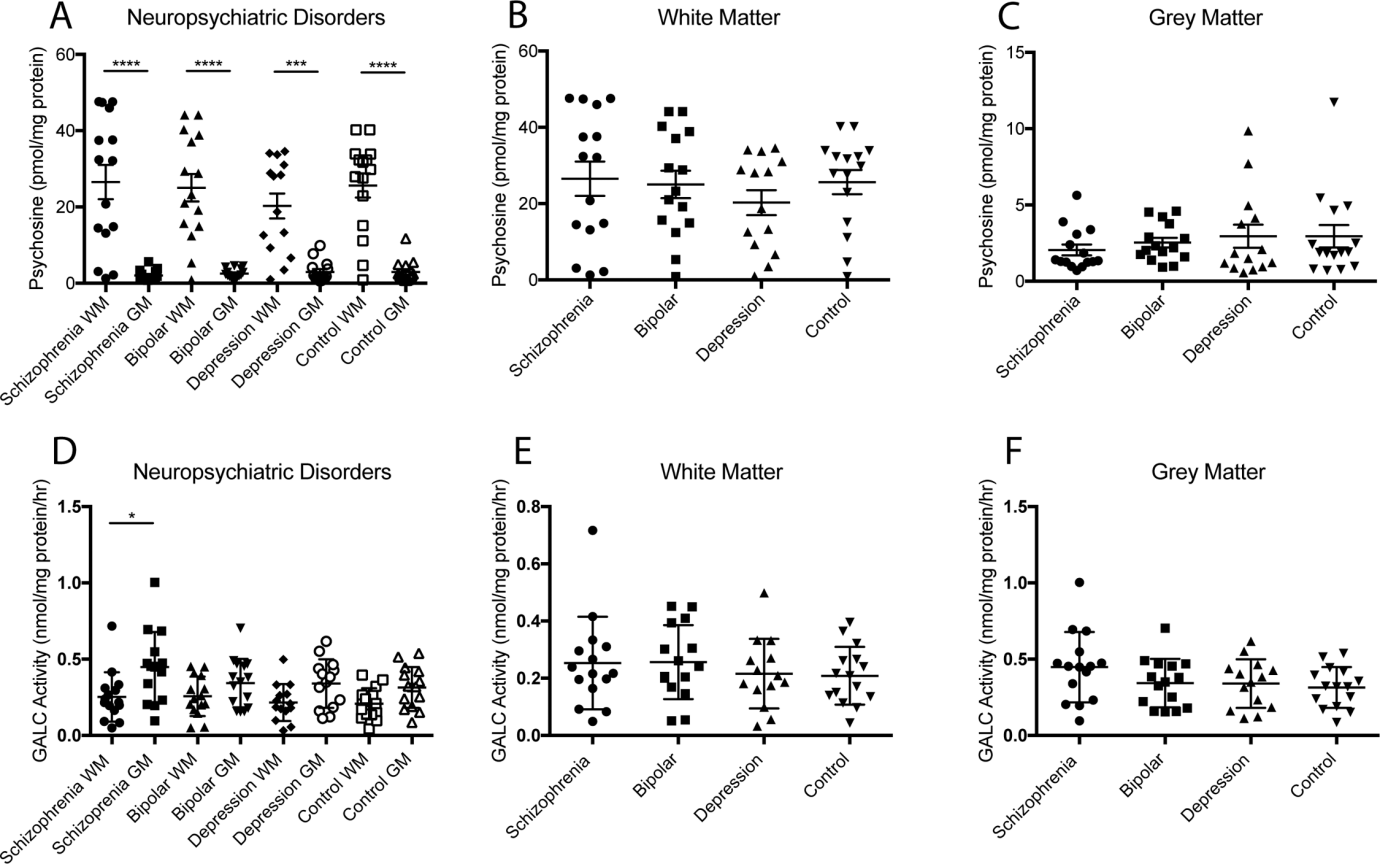
Psychosine content and GALC activity in neuropsychiatric cohort. Psychosine content and GALC activity was measured in white (WM) and gray (GM) matter of the cortex in patients diagnosed with schizophrenia, bipolar disorder, depression, or as age-matched healthy controls. A,D) The same robust distribution of psychosine and GALC activity was observed in this cohort as found in the older neurodegenerative cohort. B,C,E,F) No significant changes in psychosine content or GALC activity were observed between any disease state. (One way ANOVA with Tukey’s multiple comparison test, *p<0.05. **p<0.01, ***p<0.001, ****p<0.0001).

Distribution of psychosine and GALC activity among white and gray cortical matter was similar in neuropsychiatric conditions as that found in the neurodegenerative cohort. We found significantly higher psychosine content in white matter compared to gray for all conditions (Fig 2A), but between disease states there were no significant differences (Fig 2B and 2C). Also, an inverse distribution for GALC activity compared to psychosine accumulation was measured (Fig 2D). Consistent with the results from the neurodegenerative cohort, GALC activity between white and gray matter was not as disparately distributed as psychosine, reaching significance in only the schizophrenia group (Fig 2D). GALC activity across disease states did not reveal any significant changes either for any of the neurological conditions (Fig 2E and 2F).

### Aging plays a role in metabolic dysfunction of GALC activity and psychosine clearance during middle-age span

**Fig 3:**
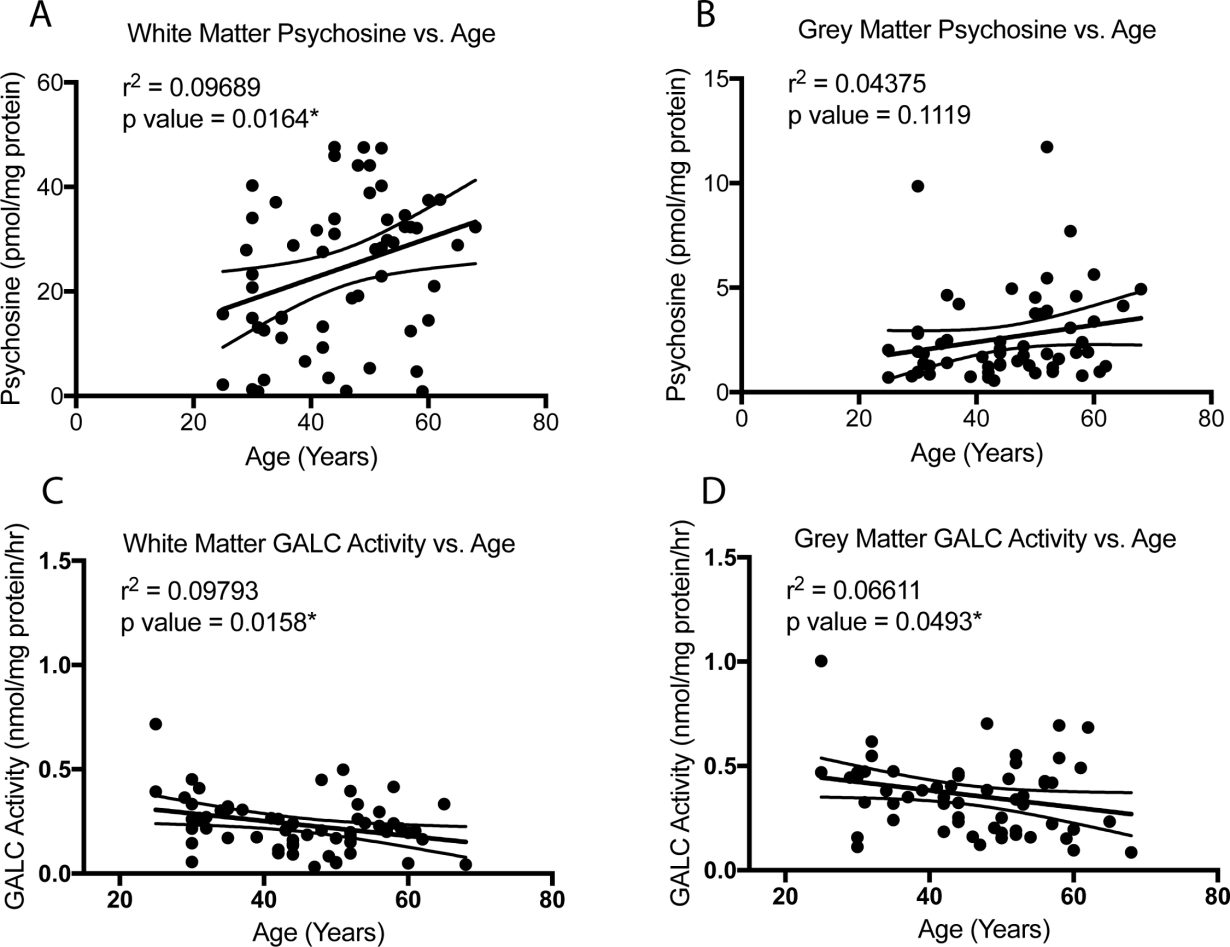
Psychosine content and GALC activity correlated with aging in mid-aged cohort. Psychosine content (A,B) and GALC activity (C,D) in cortical brain tissue from a combined cohort of mid-aged neuropsychiatric patients and healthy control patient tissue was compared to age at time of death. Linear regression revealed a significant positive correlation between psychosine content in the white matter (A) and age in this cohort, but a similar positive correlation did not reach significance in grey matter (B). However, a significant negative correlation between GALC activity and age was present in both white (C) and grey (D) matter. (Statistical test of slope significantly non-zero, *p<0.05).

GALC activity and psychosine content from all patients within the neuropsychiatric cohort were correlated against the age of each patient (Fig 3). Correlations for psychosine content and GALC activity during aging within any single disease state was not possible due to the limited sample sizes. Spanning an age range of 25 to 68 years old, linear regression showed a statistically significant, positive correlation between white matter psychosine content and age (Fig 3A). The correlation between age and gray matter psychosine, although trending, was not statistically significant (Fig 3B). Inversely, GALC activity was found to have a significant, negative correlation with age in both the white and gray matter (Fig 3C and 3D). Sex differences were not found for any of these analyses (data are identified as male and female in S3 Fig). These results suggest that like many enzymes during aging, GALC activity diminishes, allowing psychosine levels to gradually accumulate. Given prior studies finding psychosine’s acceleration of α-synuclein aggregation [8], this effect could contribute to pathological changes to α-synuclein in vulnerable patients and thereby accelerate disease onset in conjunction with other risk factors for Parkinson’s or other adult onset α-synucleinopathies.

A similar analysis was performed on the aged cohort (S4 Fig) which did not find any significant correlation between psychosine levels in age during this age span (S4A and S4B Fig). However, the aged cohort did show a significant positive correlation between GALC activity and age (S4C and S4D Fig), inverse to the trend observed in the mid-age cohort. The results within the aged cohort may be potentially confounded by the onset of neurodegeneration and demyelination that was not present in the non-symptomatic patients of the mid-age cohort. Understanding how the brain changes during the mid-age cohort before symptoms develop may be more important for understanding the pathological changes that lead to disease states. Additionally, PD patients were also plotted individually but did not show any significant correlations on their own (S4E–S4H Fig), which were also not observed in either of the other two groups when plotted individually (data not shown).

### GALC mutational analysis finds heterozygous *GALC* mutation carriers in PD patients and healthy controls but not in the AD cohort

Mutations for the Gaucher associated gene *GBA1* have been found to be one of the most significant risk factors for the development of PD [3]. To determine whether an analogous heterozygosity for *GALC* mutations may be similarly correlated with PD, DNA was isolated from tissues of the neurodegenerative cohort, the neuropsychiatric cohort and middle-aged and aged healthy controls (Table 2).

**Table 2:** GALC mutational analysis.

	Allele 1					Allele 2			
	Promoter	Exonic Variants			Synonymous	Promoter	Exonic Variants		Synonymous
AD	c.-287 G>A		p.Ile562Thr						p.Q328Q
AD	c.-287 G>A		p.Ile562Thr		p.D110D	c.-148 T>C	p.Ala21Pro	p.Asp248Asn	p.A14A/G25G
AD			p.Ile562Thr		p.S450S			p.Ile562Thr	p.S450S
AD	c.-287 G>A		p.Ile562Thr						p.Q328Q
AD					p.Q328Q				p.Q328Q
AD	c.-148 T>C	p.Ala21Pro	p.Asp248Asn		p.A14A/G25G				p.Q328Q
AD	c.-287 G>A		p.Ile562Thr						p.Q328Q
AD					p.Q328Q				p.Q328Q
AD			p.Ile562Thr		p.S450S			p.Ile562Thr	p.S450S
AD	c.-287 G>A		p.Ile562Thr						p.Q328Q
PD			p.Ile562Thr		p.S450S				p.Q328Q
PD			p.Ile562Thr		p.S450S				p.Q328Q
PD					p.Q328Q				p.Q328Q
PD			p.Ile562Thr		p.S450S				p.Q328Q
PD		p.Arg184Cys	p.Ile562Thr		p.S450S				p.Q328Q
PD		p.Arg184Cys	p.Ile562Thr		p.S450S				p.Q328Q
PD			p.Ile562Thr		p.S450S				p.Q328Q
PD	c.-148 T>C	p.Ala21Pro	p.Asp248Asn		p.A14A/G25G				p.Q328Q
PD			p.Ile562Thr		p.S450S			p.Ile562Thr	p.S450S
PD					p.Q328Q				p.Q328Q
PD	c.-287 G>A		p.Ile562Thr		p.S450S			p.Ile562Thr	p.S450S
PD		p.Arg184Cys	p.Ile562Thr	**p.Phe596Ser**	p.S450S		p.Arg184Cys	p.Ile562Thr	p.S450S
PD	c.-287 G>A	** **		p.Trp132*		c.-287 G>A			p.Q328Q
PD					p.Q328Q				p.Q328Q
PD	c.-148 T>C	p.Ala21Pro	p.Asp248Asn		p.A14A/G25G			p.Ile562Thr	p.S450S
PD			p.Ile562Thr		p.S450S			p.Ile562Thr	p.S450S
PD			p.Ile562Thr		p.S450S				p.Q328Q
PD	c.-287 G>A		p.Ile562Thr		p.D110D				p.Q328Q
PD	c.-287 G>A		p.Ile562Thr		p.D110D				p.Q328Q
PD			p.Ile562Thr		p.S450S				p.Q328Q
Control					p.Q328Q				p.Q328Q
Control		p.Arg184Cys	p.Ile562Thr		p.S450S				p.Q328Q
Control	c.-148 T>C	p.Ala21Pro	p.Asp248Asn		p.A14A/G25G	c.-287 G>A		p.Ile562Thr	p.S450S
Control			p.Ile562Thr		p.S450S			p.Ile562Thr	p.S450S
Control			p.Ile562Thr		p.S450S				p.Q328Q
Control	c.-148 T>C	p.Ala21Pro	p.Asp248Asn		p.A14A/G25G			p.Ile562Thr	p.S450S
Control			p.Ile562Thr		p.S450S				p.Q328Q
Control		p.Arg184Cys	p.Ile562Thr		p.S450S				p.Q328Q
Control	c.-287 G>A		p.Ile562Thr		p.S450S			p.Ile562Thr	p.S450S
Control	c.-148 T>C	p.Ala21Pro	p.Asp248Asn		p.A14A/G25G			p.Ile562Thr	p.S450S
Control			p.Ile562Thr		p.S450S			p.Ile562Thr	p.S450S
Control	c.-287 G>A		p.Ile562Thr						p.Q328Q
Control	c.-287 G>A		p.Ile562Thr						
Control		p.Arg184Cys	p.Ile562Thr		p.S450S		p.Arg184Cys	p.Ile562Thr	p.S450S
Control			p.Ile562Thr		p.S450S			p.Ile562Thr	p.S450S
Control			p.Ile562Thr		p.S450S			p.Ile562Thr	p.S450S
Control	c.-287 G>A				p.L5L/L133L			p.Ile562Thr	p.S450S
Control		p.Arg184Cys	p.Ile562Thr		p.S450S				p.Q328Q
Control	c.-148 T>C	p.Ala21Pro	p.Asp248Asn		p.A14A/G25G				p.Q328Q
Control			p.Ile562Thr		p.S450S				p.Q328Q
Control	c.-148 T>C	p.Ala21Pro	p.Asp248Asn		p.A14A/G25G			p.Ile562Thr	p.S450S
Control	c.-148 T>C	p.Ala21Pro	p.Asp248Asn		p.A14A/G25G			p.Ile562Thr	p.S450S
Control	c.-148 T>C	p.Ala21Pro	p.Asp248Asn		p.A14A/G25G			p.Ile562Thr	p.S450S
Control	c.-148 T>C	p.Ala21Pro	p.Asp248Asn		p.A14A/G25G			p.Ile562Thr	p.S450S
Control	c.-148 T>C	p.Ala21Pro	p.Asp248Asn	**p.Thr112Ala**	p.A14A/G25G	c.-148 T>C	p.Ala21Pro	p.Asp248Asn	p.A14A/G25G
Control					p.Q328Q				p.Q328Q
Control			p.Ile562Thr		p.S450S				p.Q328Q
Control	**c.329 -8_10 del**		p.Ile562Thr		p.S450S				p.Q328Q
Control	c.-148 T>C	p.Ala21Pro	p.Asp248Asn		p.A14A/G25G			p.Ile562Thr	p.S450S
Control			p.Ile562Thr		p.S450S				p.Q328Q
KD	c.-148 T>C	p.Ala21Pro	p.Asp248Asn		p.A14A/G25G		p.Arg184Cys	**g.30 Kb del**	
KD		p.Arg184Cys	**g.30 Kb del**				p.Arg184Cys	**g.30 Kb del**	
KD	c.-148 T>C	p.Asp248Asn	**p.Thr529Met**		p.A14A/G25G	** **	** **	**p.Tyr173Leu**	p.Q328Q
KD Late-onset	p.Ser23*	p.Arg184Cys	p.Ile562Thr		p.S450S	** **	**p.Met117Leu**	p.Ile562Thr	p.S450S
Control Infant		c.1834+5 G>C	p.Ala641Thr		p.L359L/T540T/V556V			p.Ile562Thr	p.S450S

DNA was extracted from tissues of Alzheimer’s, Parkinson’s, Krabbe, and healthy diagnosed patients. DNA was sequenced for SNPs present in *GALC*, which are listed in the table for each patient. *GALC* mutations causing severely diminished activity are listed in bold, which were confirmed in all Krabbe patients. An additional 4 patients were identified as carriers (heterozygous) for severe/mild mutations to *GALC*, 2 within patient diagnosed with Parkinson’s, and 2 within patients diagnosed without any neurological disorder.

Extracted DNA was sequenced for SNPs within *GALC* (14q31.3). No other known mutations for PD, including *GBA1*, *SCNA* or any of the known risk genes associated with PD were screened within these samples. SNPs and the common g.30Kb deletion are listed in Table 2 with severe *GALC* mutations or likely pathogenic variants that are known to lead to diminished GALC activity listed in bold. Homozygosity or compound heterozygosity for severe mutations were confirmed in all four KD patients analyzed. A second mutation was not identified on one of the KD patients; it is highly probable that this patient has a second mutation/deletion that can be missed by our assay as a limitation of the test. Additionally, three patients were identified as carriers for severe *GALC* mutations and one with a variant (p.Thr112Ala) associated with late-onset Krabbe only when in cis with the common GALC activity reducing polymorphism p.Ile562Thr [19, 20]. Two of these patient carriers for mutations p.Phe596Ser and pTrp132* were identified within patients clinically and pathologically diagnosed with PD and one carrying mutation c.329-8_10del was from a healthy control previously undiagnosed with any neurological condition. The pathogenic variant (p.Thr112Ala) was identified in another ostensibly healthy control patient. Overall, 10% (2/20) of PD patients tested were found to be a carrier for a severe *GALC* mutation compared to 0% (0/10) of Alzheimer’s patients and 6.67% (2/30) of healthy controls.

**Fig 4:**
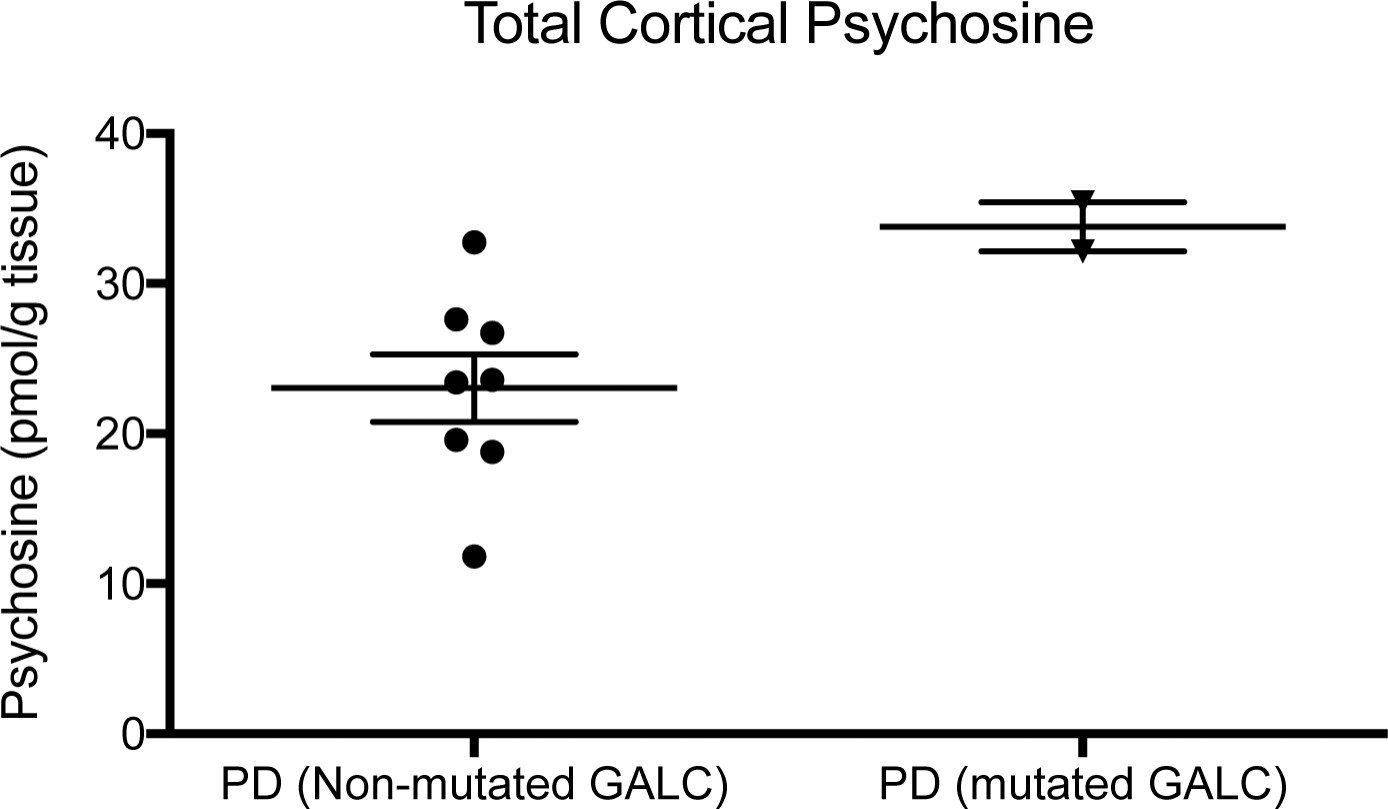
GALC mutational effect on psychosine accumulation. Severe *GALC* mutations were identified in 2 patients diagnosed with Parkinson’s disease (PD). Psychosine levels in tissue from these two patients were elevated compared to that measured in tissue from PD patients without a severe *GALC* mutation.

The presence of mutations p.Phe596Ser and pTrp132* coincided with an increase in total cortical levels of psychosine in both PD patients (Fig 4). Tissues analyzed in Fig 4 came from the same brain repository of fixed tissue, therefore, brain region and the handling of tissue post-mortem was consistent. Measurement of GALC activity in these tissues was not feasible due to the denaturing conditions exerted by the fixative. Statistical significance of psychosine’s elevation was not feasible due to the sample size of only 2 PD patients with mutations. Also, due to the difference in tissue handling of fixation versus fresh-frozen tissue used in Fig 1, these values could also not be compared to the values measured in the cohorts from Fig 1. However, these results further support that severe *GALC* mutations coincide with higher psychosine levels in the aged adult brain. Taken together with our previous findings that increased psychosine can induce α-synuclein aggregation [8], this begins to elucidate the mechanism by which *GALC* mutations can be a risk factor for PD [18].

Patients diagnosed as healthy but containing a severe *GALC* mutation or pathogenic variant, were 58 and 71 years of age respectively and showed normal pigmentation within the substantia nigra (per pathology reports). However, α-synuclein accumulation had not been examined, and given both patients’ relatively younger ages, incidental Lewy body disease or an early not-yet clinically manifested α-synuclein pathology may be present. The level of psychosine and GALC activity measured within these samples did not show any significant increase or reduction compared to the mean of the healthy cohorts.

### α-Synuclein pathology is associated with Krabbe disease and *GALC* mutations

**Fig 5:**
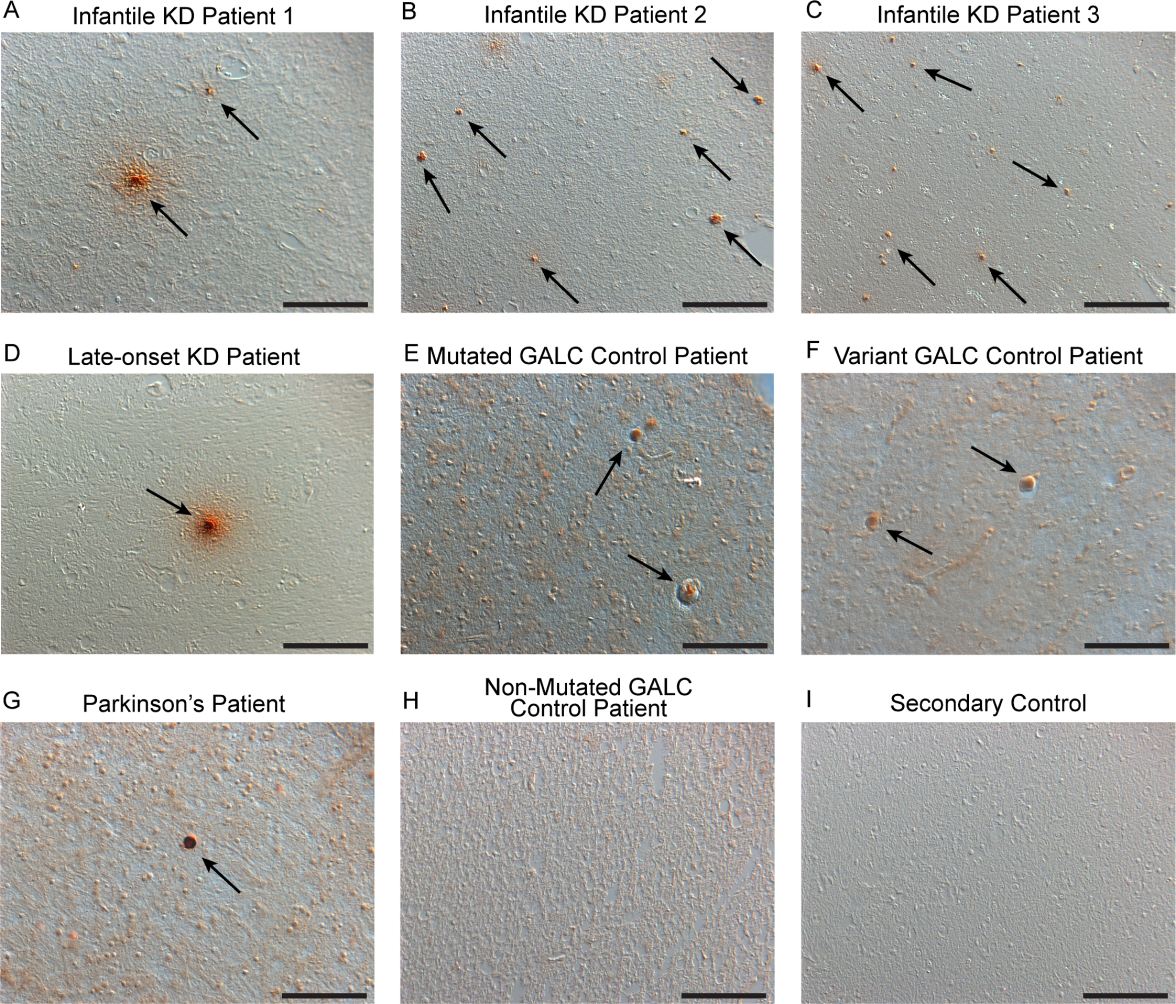
Immunohistological staining for α-synuclein. Immunohistological staining for α-synuclein was performed in brain tissue from 3 infantile KD patients (A-C), a late-onset KD patient (D), 2 patients found to be *GALC* mutation/variant carriers with no neurological disorders previously diagnosed (E,F), a Parkinson’s patient (G), and a healthy control with no *GALC* mutations (H). Accumulations of α-synuclein were observed in all Krabbe patients (A-D), the Parkinson’s brain (G), and also within the brains of *GALC* mutation/variant carriers (E,F). Accumulations in the infantile brains were abundant but smaller in size than the Lewy bodies observed in the Parkinson’s brain and the α-synuclein accumulations found in late-onset KD and *GALC* mutation carrier tissue. No significant α-synuclein accumulation was observed in the healthy diagnosed patient without GALC mutations (H). A secondary control experiment confirmed the specificity of the secondary antibody (I). Scale Bars: 100 μm.

Pathological changes in α-synuclein accumulation were examined in frontal cortex tissue from KD, PD and healthy diagnosed tissue (with and without *GALC* mutation heterogeneity) by immunohistochemically staining for α-synuclein (Fig 5). Accumulations of α-synuclein were detected in three different patients with infantile KD and in one late-onset KD patient (Fig 5A–5D, arrows). Accumulations in infantile cases (Fig 5A–5C, arrows) were abundant but typically smaller than that observed in a Lewy body of Parkinson’s patients (Fig 5G, arrow), which is depicted as a representative example of α-synuclein accumulation. α-Synuclein accumulations found in the late-onset KD brain were larger and closer in size to the PD brain (Fig 5D and 5G, arrows), potentially due to the longevity of the patient and timeframe for aggregates to accumulate. Examining brain tissue from the two patients carrying a severe *GALC* mutation or pathogenic variant, yet undiagnosed with neurological pathology, found evidence of α-synuclein accumulation in both brains (Fig 5E and 5F, respectively). Conversely, a healthy control patient containing no severe *GALC* SNPs was not found to contain detectable accumulations of α-synuclein (Fig 5H). While a significant quantitative correlation between the presence of α-synuclein accumulation and GALC mutations cannot be determined from these number of samples, finding evidence of α-synuclein accumulation within GALC mutation carriers suggests a correlation is possible, the physiological consequence of which is yet to be determined. Additionally, the specificity of the secondary antibodies used was also confirmed on frontal cortex tissue of an aggregated α-synuclein containing PD patient (Fig 5I).

## Discussion

Many lysosomal storage diseases are compounded by protein aggregates [25–27], including KD which includes neuronal inclusions composed of at least in part by α-synuclein [8]. The developmental pattern of these neuronal inclusions within the KD mouse (Twitcher) is similar to the Braak staging that describes Lewy body progression within PD brains [28]. Brain regions in the Twitcher that are highly affected by protein inclusions are also found to be highly enriched in psychosine [8], suggesting that psychosine may be a contributor driving this pattern of aggregation in vivo. Evidence for psychosine’s role in α-synuclein aggregation is further supported by psychosine accelerating α-synuclein aggregation *in vitro* [8] and by NMR experiments that show a direct interaction between psychosine and α-synuclein that promotes an aggregation prone configuration in α-synuclein (Abdelkarim, Marshall, Gaponenko & Bongarzone, under review).

Whether regional distributions of psychosine within the adult brain may influence the accumulation of α-synuclein is unknown. This study measured the content of psychosine in multiple brain regions of the adult brain, finding that psychosine follows a robust pattern of distribution, enriched within the white matter of the cortex and the substantia nigra in comparison to the cortical gray matter and caudate. Lewy body accumulation within the substantia nigra is a hallmark of PD [29] and the cortical white matter is a region that commonly undergoes pathological changes in PD as well [30], especially in patients with *GBA1* gene mutations [31]. Importantly, psychosine concentrations within the cortex of PD brains were higher than the levels measured in age-matched healthy tissue, and particularly in PD patients carrying mutations p.Phe596Ser and pTrp132* in the *GALC* gene. This correlation provides evidence for the first time that psychosine levels and some mutations in the GALC gene may also contribute to the distribution and/or accumulation of aberrant α-synuclein in an adult α-synucleinopathy such as PD.

Aging remains the largest risk factor for developing idiopathic PD [32], and was shown in this study to be correlated with psychosine accumulation and reductions in GALC activity during a middle-age timespan. While psychosine-associated α-synuclein pathology may not be the root cause of PD, this age/genotype dependent accumulation of a neurotoxin known to accelerate α-synuclein fibrillization in patients affected by Krabbe disease may contribute to increase pathology in certain vulnerable patients in conjunction with other PD risk factors. An age-dependent loss in psychosine hydrolysis may remain subclinical and silent in most patients, only inducing or accelerating neuropathological changes in at-risk individuals. Due to psychosine’s role in demyelination in affected Krabbe patients, our study does not exclude that age-related increases of psychosine may also contribute to myelin vulnerability in the aged brain and/or other demyelinating conditions [9].

In addition to biochemical evidence of PD’s connection to psychosine metabolism, this study also revealed a genetic correlation. It has been found that patients with Gaucher’s disease have a nearly 20-fold increased risk of developing PD in their life-time [33], with *GBA1* heterozygotes also having an increased risk of PD [34] and an association with earlier onset and more severe forms of PD [6, 7]. The *GALC* gene has now recently been identified as a risk loci for PD [18], coinciding with our results that found 10% (2/20) of PD patients tested to be a carrier for a severe *GALC* mutation (i.e. mutations p.Phe596Ser and pTrp132*) compared to 0% (0/10) of Alzheimer’s patients and 6.67% (2/30) of healthy controls. The finding that carriers were only identified in PD rather than AD patients provides more evidence that *GALC* mutations, and deregulation of psychosine content, could play a role in α-synuclein aggregation rather than neurodegeneration in general. However, the smaller sample size of AD patients available in this study is unable to fully exclude the possibility of GALC dysregulation playing a larger role in neurodegeneration vs specifically PD or α-synucleinopathies. Still, to our knowledge, the GALC gene has only been identified to date as a risk loci for PD [18] and not for other neurodegenerative disorders.

There have been no previous reports to date of increased incidence of PD in patients with KD or in relatives of affected individuals as has been reported in Gaucher’s disease [35, 36]. In general, infantile Krabbe patients do not survive beyond the third year of life, precluding any association with PD symptoms to emerge. However, genetic studies in late-onset KD patients has provided some evidence that reduced GALC enzymatic activity can result in delayed and moderate neuropathology, with moderate development of dementia and neurodegeneration [37–39]. In one study, half of patients with adult onset KD were heterozygous for the 30-kb deletion (C502T) in GALC, a mutation that eliminates all enzymatic activity [40]. Therefore, ≤50% GALC activity was not clinically apparent until adulthood. SNPs that result in milder reductions to GALC activity may remain silent for longer, only manifesting in old-age and potentially with unique presentations. Also, it has been proposed that the haplotype effect (variants on the same chromosome) plays an important role in late-onset KD [19]. No KD patient has been found to be homozygous for two common GALC activity reducing variants, (p.Thr112Ala and p.Tyr319Cys), in the NYS newborn screening referral population; however, they are considered disease-causing alleles when in cis with the common enzyme activity reducing (60–70%) p.Ile562Thr polymorphism or in trans with a severe allele [20, 41]. Again, the contribution of *GALC* heterozygosity to developing PD may only be relevant in patients with additional genetic and environmental risk factors for PD, acting only as a disease modifier, rather than the primary pathophysiological cause.

An important finding of this genetic screen was the identification of severe and mild *GALC* mutations in two patients diagnosed without any neurologically abnormalities (per pathology reports). PD had been excluded based on normal pigmentation in the substantia nigra; however, no examination had been performed for α-synuclein pathology. Here, we performed immunohistochemical staining for α-synuclein, finding evidence of α-synuclein accumulation that was not present in tissue from healthy control patients without a severe or mild *GALC* mutation. While the number of patients and amount of tissue available was not sufficient for a quantitative analysis, these findings suggest that reduced GALC activity due to mutations in the *GALC* gene of these two patients could have facilitated an increased formation of Lewy-body like inclusions in the brain. Had these patients lived longer (ages at death: 58 and 71 years), symptoms may have become clinically apparent. The amount of aberrant α-synuclein observed may also have been benign and remained so due to a lack of compounding risk factors. However, in patients diagnosed with KD, the number of α-synuclein inclusions was increased, supporting the role of psychosine in α-synuclein aggregation.

Psychosine can be monitored non-invasively from peripheral blood [41, 42], and its peripheral level correlates to the accumulation present within the central nervous system [42]. Therefore, confirming psychosine’s role in the progression of PD in certain patient populations could provide a new, easily measurable biomarker. This study also found that *GALC* mutations could potentially be used to identify patients at risk for PD, such as the link found for *GBA1* mutations. Additional validation for psychosine and *GALC* mutations in larger cohort studies will strengthen this area. Overall, these results are an important first step for identifying the relevance of psychosine metabolism outside of KD and its potential impact on other α-synucleinopathies such as PD and during aging of the human brain.

## Supporting information

S1 Fig Tyrosine hydroxylase content in SN of neurodegenerative cohort. A) Dopaminergic neuronal content was estimated via tyrosine hydroxylase (TH) immunoblotting in SN extracts from the neurodegenerative cohort, which showed a decreased level in PD patients. B) TH content, normalized to actin, was correlated with psychosine levels, finding no significant correlation. Significance test of slope significantly non-zero.
(TIF)

S2 Fig Mass spectrometry analysis of psychosines. Lipid extracts from substantia nigra (SN) or cortex were processed for detection of glucopsychosine and psychosine as described and analyzed by tandem mass spectrometry. A) A mixture of glucopsychosine and psychosine standards shows separation of peaks for each psychosine and confirmatory spectra (insets); B-D) Analyses in lipid extracts from SN from Parkinson’s disease (B), Alzheimer’s disease (D) and from cortex from a Krabbe’s disease (C) cases identified psychosine as the only psychosine species present.
(TIF)

S3 Fig Male and female distribution of psychosine content and GALC activity correlation with aging in mid-aged cohort. Data from Fig 3 is reproduced with data points identified as male or female.
(TIF)

S4 Fig Psychosine content and GALC activity correlation within the neurodegenerative aged cohort. Psychosine content (A,B) and GALC activity (C,D) in cortical brain tissue from a combined cohort of aged neurodegenerative patients and healthy control patient tissue was compared to age at time of death. Linear regression revealed no correlation between psychosine content in the white matter (A) or grey matter (B) and age in this cohort. However, a significant positive correlation between GALC activity and age was present in both white (C) and grey (D) matter. (Statistical test of slope significantly non-zero, *p<0.05).
(TIF)

S1 Table Raw data file. Data collected during this project and presented in the results section is included here.
(XLSX)
