# Supplementary figures and images for: Analysis of age-related changes in psychosine metabolism in the human brain
Source: PLoS One. 2018 Feb 26;13(2):e0193438. doi: 10.1371/journal.pone.0193438 (PMC5826537; doi:10.1371/journal.pone.0193438)

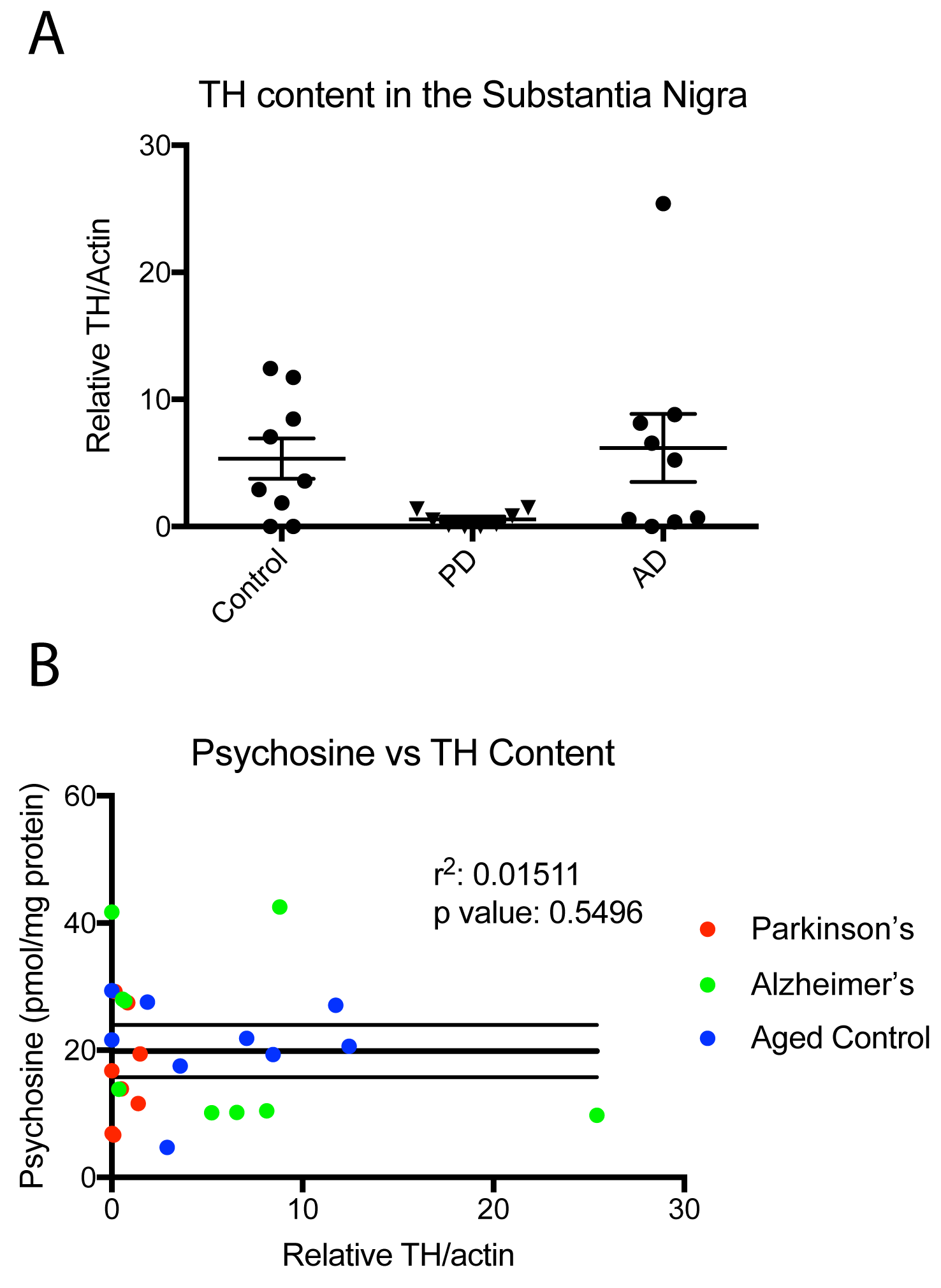

Supplement: S1 Fig — A) Dopaminergic neuronal content was estimated via tyrosine hydroxylase (TH) immunoblotting in SN extracts from the neurodegenerative cohort, which showed a decreased level in PD patients. B) TH content, normalized to actin, was correlated with psychosine levels, finding no significant correlation. Significance test of slope significantly non-zero. (TIF) [file pone.0193438.s001.tif]

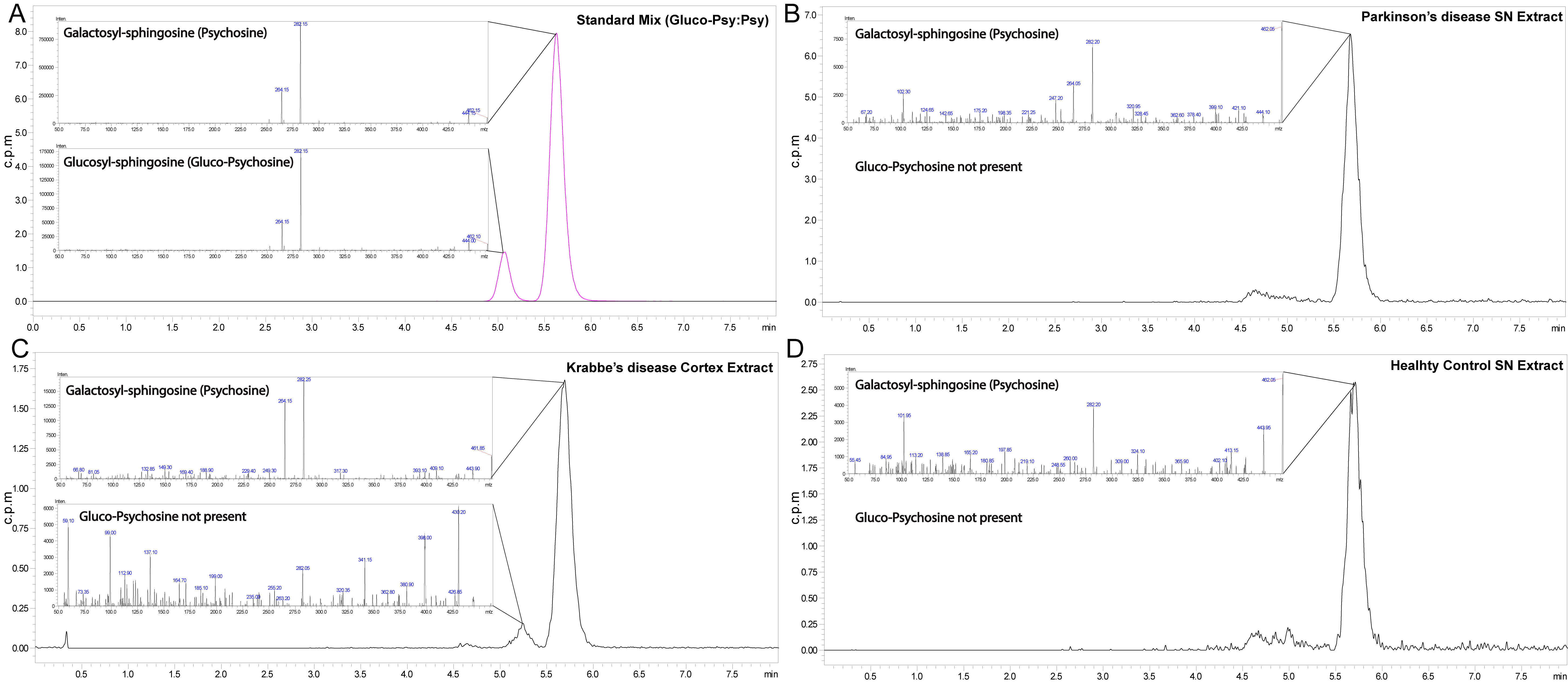

Supplement: S2 Fig — Lipid extracts from substantia nigra (SN) or cortex were processed for detection of glucopsychosine and psychosine as described and analyzed by tandem mass spectrometry. A) A mixture of glucopsychosine and psychosine standards shows separation of peaks for each psychosine and confirmatory spectra (insets); B-D) Analyses in lipid extracts from SN from Parkinson’s disease (B), Alzheimer’s disease (D) and from cortex from a Krabbe’s disease (C) cases identified psychosine as the only psychosine species present. (TIF) [file pone.0193438.s002.tif]

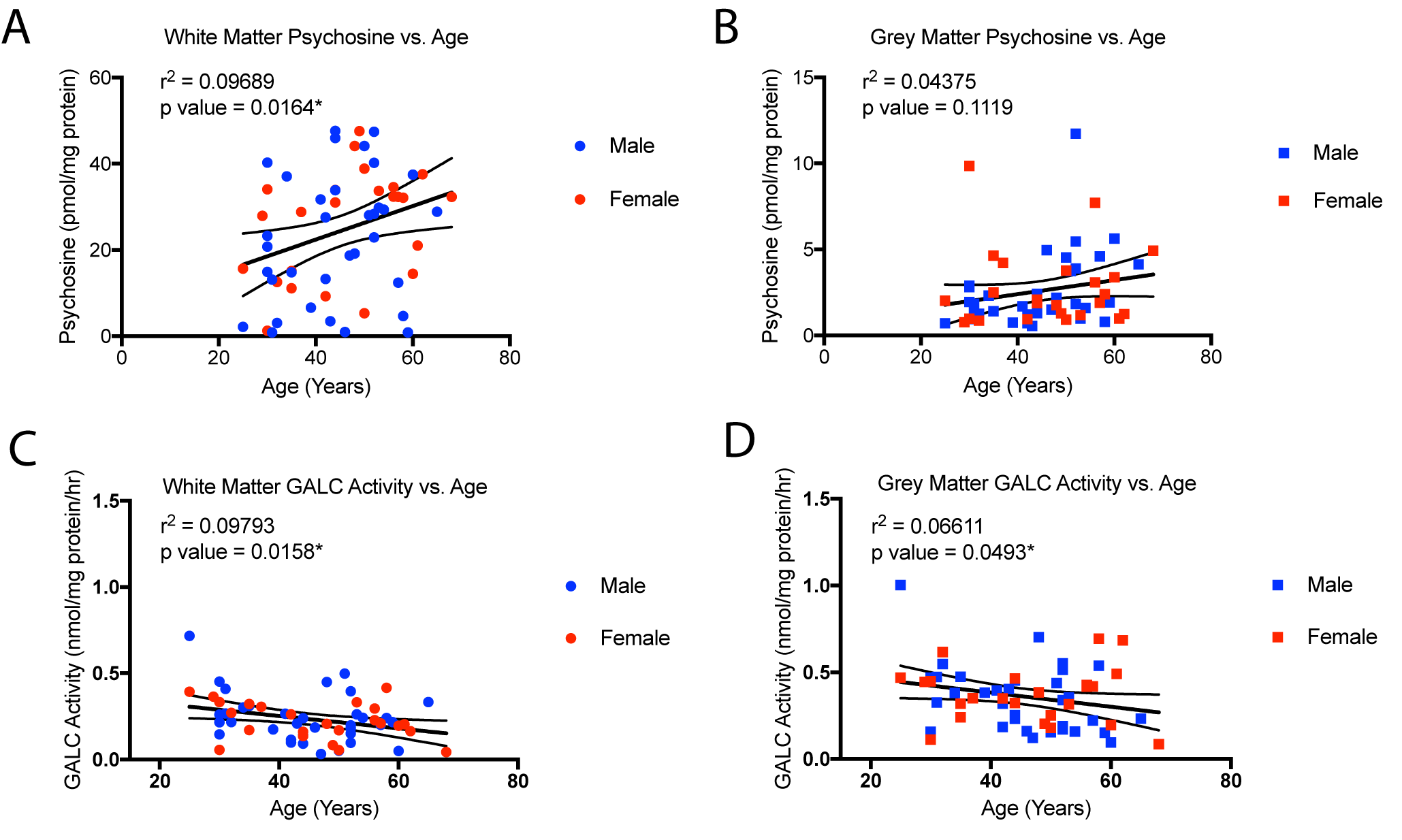

Supplement: S3 Fig — Data from Fig 3 is reproduced with data points identified as male or female. (TIF) [file pone.0193438.s003.tif]

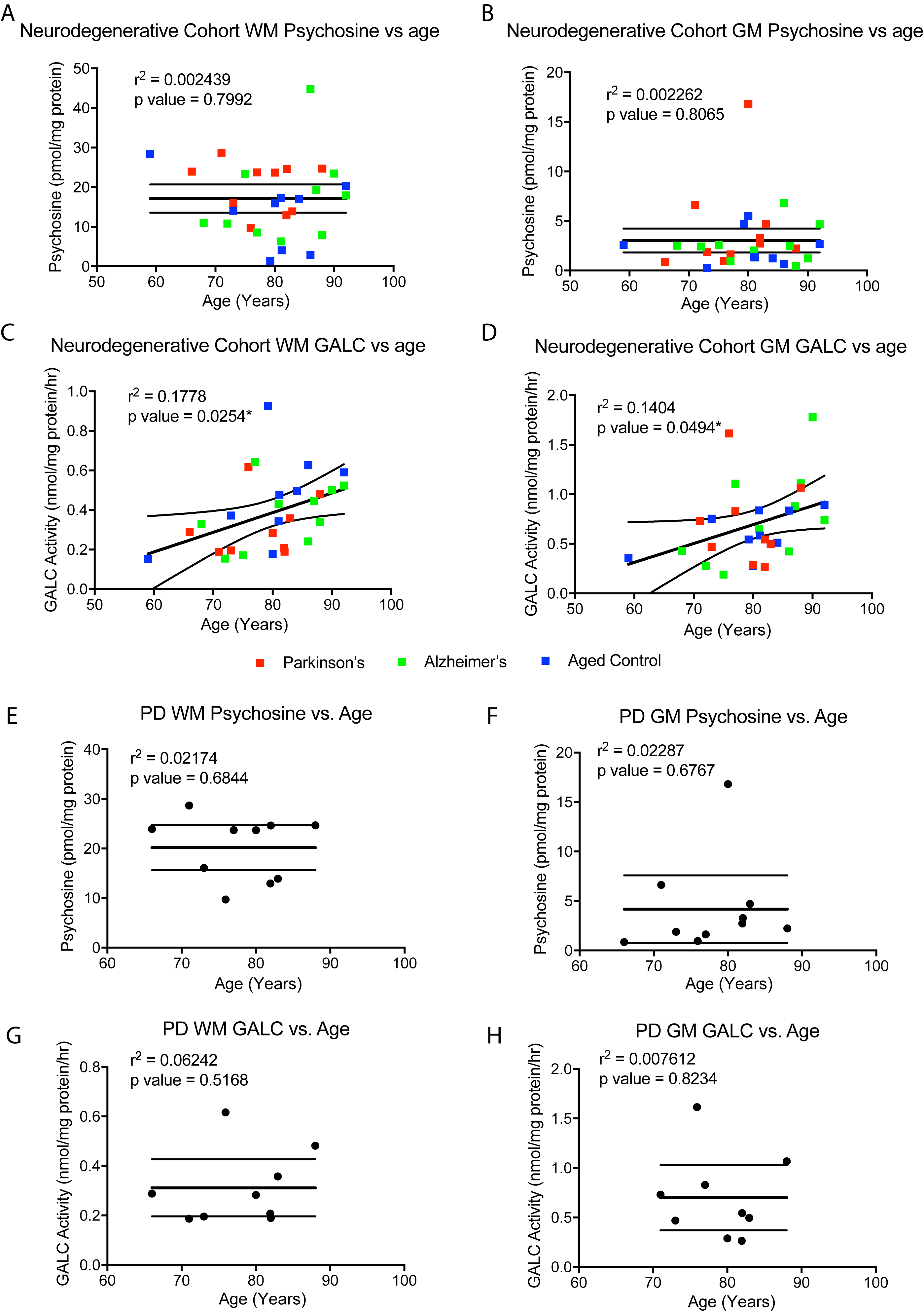

Supplement: S4 Fig — Psychosine content (A,B) and GALC activity (C,D) in cortical brain tissue from a combined cohort of aged neurodegenerative patients and healthy control patient tissue was compared to age at time of death. Linear regression revealed no correlation between psychosine content in the white matter (A) or grey matter (B) and age in this cohort. However, a significant positive correlation between GALC activity and age was present in both white (C) and grey (D) matter. (Statistical test of slope significantly non-zero, *p<0.05). (TIF) [file pone.0193438.s004.tif]
